# Health literacy status and associated factors among residents in Anhui Province, China: a cross-sectional study

**DOI:** 10.3389/fpubh.2024.1493682

**Published:** 2025-01-09

**Authors:** Yuansheng Fu, Jianrong Xie, De Xu, Yuanrui Xia, Zhimin Wang, Yinguang Fan

**Affiliations:** ^1^Department of Health Education, Anhui Provincial Center for Disease Control and Prevention, Hefei, China; ^2^Department of Epidemiology and Biostatistics, School of Public Health, Anhui Medical University, Hefei, China

**Keywords:** health literacy, residents, associated factors, health education, health promotion

## Abstract

**Background:**

Health literacy (HL) is a critical determinant of health outcomes. Improving HL stands as one of the most essential, cost-effective, and efficacious strategies for enhancing the overall health of the population. This study aims to analyze the status of HL among urban and rural residents in Anhui Province, explore the associated factors, and provide a scientific basis for the formulation of targeted health education and promotion strategies.

**Methods:**

A cross-sectional survey was conducted on 16,080 non-collective residents in Anhui Province from July to September 2022. Participants were selected using a multi-stage stratified random sampling method. HL was measured using the Chinese Citizen Health Literacy Questionnaire. Multivariable logistic regression analysis was performed to identify factors associated with adequate HL.

**Results:**

Overall, 29.60% of residents in Anhui Province had adequate HL. The proportion of adequate HL in the different cities ranged from 22.42 to 38.73%. Multivariable logistic regression analysis revealed that the proportion of individuals with adequate HL was higher for males than for females (adjusted odds ratio [aOR], 1.200; 95% confidence interval [CI], 1.086–1.326); married was higher than unmarried (aOR = 1.195, 95% CI: 1.021–1.398). Compared with illiterate/less literate, the aOR values for primary school, junior high school, senior high school, and college or above were 1.690 (1.326–2.155), 3.467 (2.760–4.356), 7.033 (5.516–8.968), and 17.895 (13.948–22.959), respectively; compared with the age group of 65–69 years, the aOR values for the age groups 15–24, 25–34, 35–44, 45–54 and 55–64 years were 2.384 (1.774–3.202), 2.598 (2.049–3.294), 2.862 (2.267–3.615), 2.135 (1.697–2.685), and 1.468 (1.157–1.863), respectively; compared with farmers, the aOR values were 1.244 (95% CI, 1.081–1.432) for technical/professional, 1.121 (95% CI, 1.003–1.254) for commercial/service, and 1.329 (95% CI, 1.163–1.518) for other occupations.

**Conclusion:**

Residents of Anhui Province exhibit relatively low levels of HL, with notable disparities observed among different education levels, age groups, genders, and marital statuses. It is essential for health policymakers and public health practitioners to develop targeted health education and promotion strategies tailored to distinct subpopulations of residents.

## Introduction

Health literacy (HL) refers to an individual’s ability to acquire, understand, and process basic health information and services, as well as to utilize this knowledge to make informed decisions that maintain and promote their health (1). Since its introduction in the 1970s, this concept has garnered considerable global attention, particularly within the field of public health (2). As an essential determinant of health, HL not only directly impacts residents’ life expectancy and quality of life but also serves as a crucial indicator for evaluating the level of health services in a given region (3, 4). Low HL is associated with higher-risk behaviors, increased readmission rates, and poorer health outcomes (3, 4). It can result in poorly managed chronic diseases, diminished healthcare quality, increased morbidity and mortality, and heightened healthcare expenditures (5–7). Therefore, enhancing the public’s HL is a crucial strategy for improving the overall health condition of the population.

In China, the research on HL started relatively late, and the problems are particularly prominent. In recent years, with the rapid economic and social development coupled with the intensification of population aging, the incidence of non-communicable diseases (such as diabetes, hypertension, etc.) has been continuously increasing, and the management and prevention of these diseases have put forward higher requirements for residents’ HL. Although the HL level of Chinese residents has improved, it remains relatively low overall. For example, in 2021, only 25.4% of Chinese residents had adequate HL, which means that about three-quarters of the population lacked basic health knowledge and skills (8). In addition, there are significant differences in the level of HL among different regions in China, with the eastern coastal areas performing better than the central and western regions, and cities performing better than rural areas (9, 10). Given that the HL level of residents serves as a comprehensive evaluation indicator reflecting both the level of economic and social development and the health status of the general population, China attaches great importance to enhancing HL, considering it a prerequisite for improving the health of the entire populace (11, 12).

Anhui Province is located in the eastern part of China. It is an integral part of the country’s most dynamic region—the Yangtze River Delta. It faces many similar challenges to the other areas of improving HL. To better understand the HL status and its associated factors among urban and rural residents in Anhui Province, this study conducted a cross-sectional survey. Through this study, we aim to identify the key factors associated HL among residents in Anhui Province, uncover weaknesses, and propose targeted intervention measures to improve their HL levels, ultimately improving their health status.

## Methods

### Study design and subjects

This cross-sectional study was conducted in Anhui Province, China, from July to September 2022. The target subjects of this study are permanent residents aged 15–69 years who live in non-collective housing in Anhui Province, excluding residents living in hospitals, nursing homes, military bases, prisons, and college dormitories. The permanent population refers to subjects who have lived in the local area for more than 6 months in the past year, regardless of whether they have a local registered residence or not.

The questionnaire survey was conducted through face-to-face interviews. All investigators received professional training and were qualified to engage in investigation activities after passing examinations. Written informed consent was obtained from each participant before starting the survey. This study was approved by the ethical review committee of the Anhui Provincial Center for Disease Control and Prevention.

### Sampling methods

The sample size for each county (district) was calculated using the formula $N=\frac{u_{\alpha }^{2}\times p\times \left(1-p\right)}{\delta^{2}}\times \mathrm{deff}$ .Based on the HL level of Anhui Province in 2021, which was 28.57% (13), *p* was set as 0.2857; the allowable relative error was 15%; the allowable absolute error *δ* = 0.2857 × 0.15 = 0.0429; *μα* = 1.96, *deff* = 1, the minimum sample size for each county was finally calculated as 426. Taking into account the invalid questionnaires and rejection rates, we expanded the sample size to 620 individuals per county (district).

**Figure 1:**
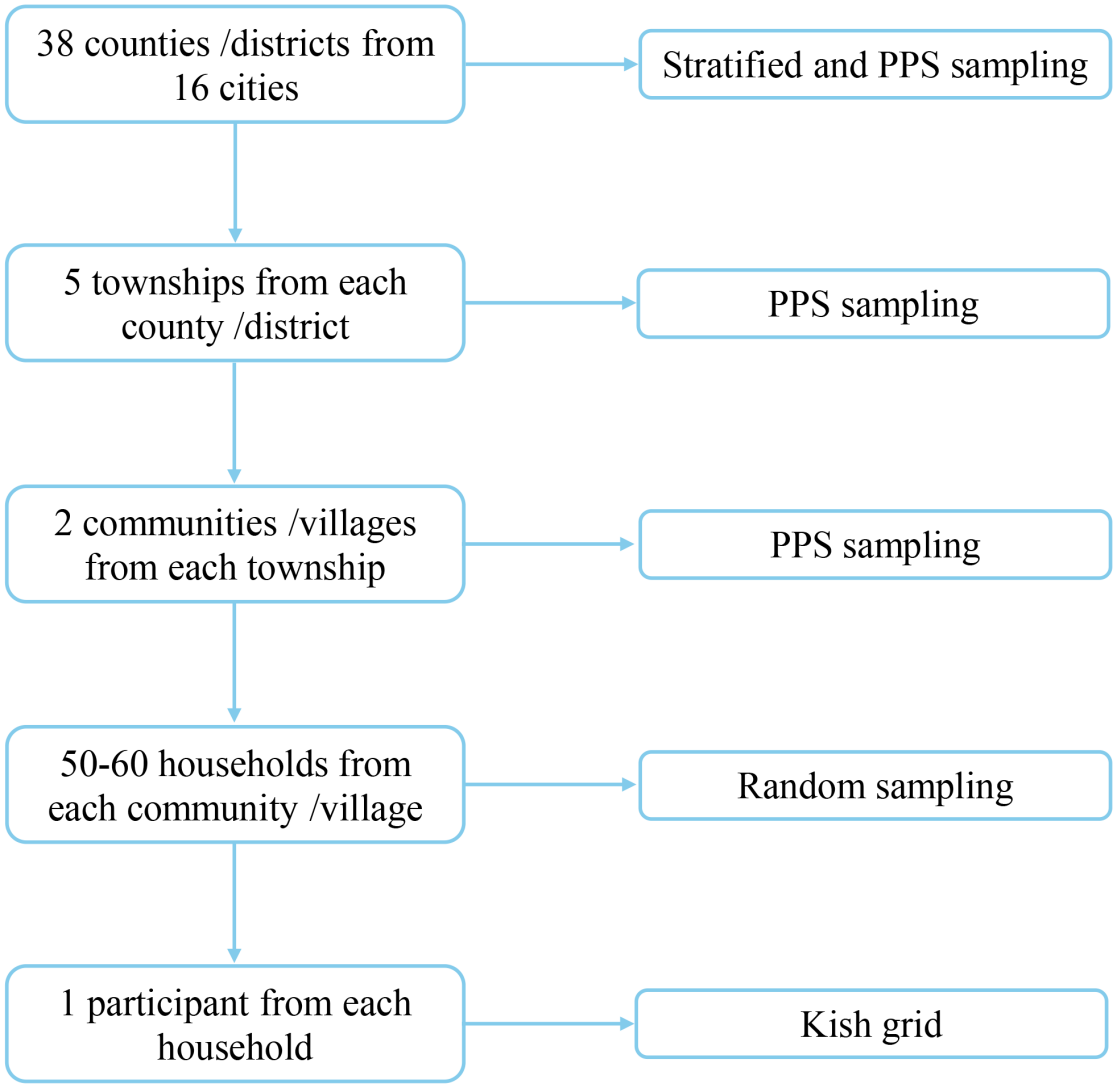
Flow chart of sampling.

A multi-stage stratified probability proportional to size (PPS) sampling was used to select survey subjects (14). The whole sampling was divided into five stages: (a) a total of 38 counties (districts) were selected from 16 cities using the method of stratified sampling and PPS sampling; (b) five townships were selected from each of county (district) using the method of PPS sampling; (c) two communities (villages) were selected from each of township using the same method as in the previous stage; (d) 50–60 households were randomly selected from each community (village); and (e) one participant from each household was selected using a Kish grid (15) (Figure 1). Finally, 17,349 questionnaires were collected, of which 16,080 were qualified and included in the final analyses.

### Study measures

The questionnaire used in this study consists of two parts: general demographic information and HL assessment. Demographic information was collected through self-report of respondents, mainly including gender, age, ethnicity, place of residence, occupation, education level, marital status, smoking status, chronic conditions, and self-rated health status.

The Chinese Health Literacy Scale, which was developed by the Chinese Center for Health Education, was used to assess HL (16). This scale was designed based on the “Chinese Resident Health Literacy—Basic Knowledge and Skills (2015 Edition)” issued by the National Health Commission of the People’s Republic of China (17). The overall Cronbach’s alpha of this scale was 0.95, and the Spearman-Brown coefficient was 0.94 (18).

This scale consists of 50 items, divided into three dimensions: basic knowledge and attitudes (BKA), healthy lifestyles and behaviors (HLB), and health-related skills (HRS). It also covers six aspects: scientific views of health, infectious diseases prevention and treatment, chronic diseases prevention and treatment, safety and first aid, primary medical care, and health information (19). The distinction between the three dimensions and six aspects of HL lies in their respective scopes and emphases. The three dimensions concentrate on establishing a comprehensive framework for HL, encompassing health knowledge, behavior, and skills. In contrast, the six aspects delve into more detailed assessments, targeting the literacy levels regarding specific health issues.

There are three types of questions on the scale: true or false (one point for a correct answer), single answer (one point for a correct answer), and multiple answers (a multiple-choice question with more than one correct answer, where two points are awarded only if all correct answers are selected, and no points are given for incorrect, missing, or excessive answers). The total score of the scale is 66 points, with a score of 53 (80% of 66) or higher considered to have adequate HL, and a score of 0–52 considered to have limited HL (19). The HL level refers to the proportion of people with adequate HL in the total population. The criterion for judging adequate HL in each dimension or aspect is to achieve a score of ≥80% of the total score for that dimension or aspect (19).

### Statistical analysis

Statistical analyses were performed using R software (version 4.2.2). QGIS software (version 3.36) was used to map the geographic distribution. Descriptive statistics, including the frequency, percentage, mean and standard deviation (SD), were reported. Chi-square tests were used to compare the HL levels among different characteristic groups. Variables with a *p*-value <0.05 in univariate analysis were selected and further explored in a multivariable logistic regression model to evaluate the factors associated with adequate HL. Odds ratios (ORs) and 95% confidence intervals (95% CIs) were calculated. A two-tailed *p* < 0.05 was considered statistically significant. To control for selection bias introduced by the sampling procedure, the sample was weighted and adjusted based on the 2020 Chinese census data (20). The final weight = base weight × non-response adjustment weight × post-stratification adjustment weight. Among them, the base weight, also known as the sampling design weight, is the reciprocal of the sampling probability of the sample unit; The non-response adjustment weight refers to the correction of the base weight to correct the bias caused by non-response when there is non-response; The post-stratification adjustment weight refers to the stratification according to key demographic indicators such as gender and age. Within each adjustment layer, the post-stratification adjustment coefficient is equal to the population of the provincial population data in that layer divided by the weighted sample number of that layer (19).

## Results

### Basic characteristics

The characteristics of the participants were shown in Table 1. A total of 17,349 individuals were surveyed, and 16,080 valid questionnaires were collected, with an effective response rate of 92.45%. More than half of the participants were female (54.4%), and male: female ratio was 1:19. The average age of the participants was 43.03 ± 14.94 years. The majority of the participants were Han ethnicity (98.99%), and 52.44% were lived in rural areas. Over one-third of the participants had a Junior high school level of education, and 43.32% were farmers. Most participants were married (76.26%), and non-smoking (71.44%). In addition, 77.52% of the participants did not have chronic diseases, and 33.44% rated their health status as “relatively good.”

**Table 1:** Association between HL level and basic characteristics.

Variable	*N* (%)	Health literacy		*χ^2^*	*p* value
		Adequate HL (%)	Limited HL (%)		
Place of residence				0.111	0.739
Urban	7,648 (47.56)	2,213 (28.94)	5,435 (71.06)		
Rural	8,432 (52.44)	2,460 (29.17)	5,972 (70.83)		
Gender				44.514	<0.001
Male	7,342 (45.66)	2,325 (31.67)	5,017 (68.33)		
Female	8,738 (54.34)	2,348 (26.87)	6,390 (73.13)		
Age group (years)				1328.997	<0.001
15–24	2,191 (13.63)	919 (41.94)	1,272 (58.06)		
25–34	2,962 (18.42)	1,326 (44.77)	1,636 (55.23)		
35–44	2,923 (18.18)	1,090 (37.29)	1833 (62.71)		
45–54	3,735 (23.23)	816 (21.85)	2,919 (78.15)		
55–64	2,880 (17.91)	416 (14.44)	2,464 (85.56)		
65–69	1,389 (8.64)	106 (7.63)	1,283 (92.37)		
Ethnicity				2.407	0.121
Han	15,918 (98.99)	4,617 (29.00)	11,301 (71.00)		
Others	162 (1.01)	56 (34.57)	106 (65.43)		
Education level				3006.156	<0.001
Illiterate/Less literate	2002 (12.45)	98 (4.90)	1904 (95.10)		
Primary school	2,767 (17.21)	290 (10.48)	2,477 (89.52)		
Junior high school	5,658 (35.19)	1,344 (23.75)	4,314 (76.25)		
Senior high school	2,760 (17.16)	1,073 (38.88)	1,687 (61.12)		
College or above	2,893 (17.99)	1868 (64.57)	1,025 (35.43)		
Occupation				1411.747	<0.001
Farmers	6,966 (43.32)	1,031 (14.80)	5,935 (85.20)		
Technical/ Professional	1970 (12.25)	1,034 (52.49)	936 (47.51)		
Students	1,480 (9.20)	584 (39.46)	896 (60.54)		
Commercial / Service	3,826 (23.79)	1,374 (35.91)	2,452 (64.09)		
Others	1838 (11.43)	650 (35.36)	1,188 (64.64)		
Marital status				307.914	<0.001
Unmarried	2,902 (18.05)	1,207 (41.59)	1,695 (58.41)		
Married	12,262 (76.26)	3,307 (26.97)	8,955 (73.03)		
Separated/Divorced/Widowed	916 (5.70)	159 (17.36)	757 (82.64)		
Smoking status				8.682	0.013
Smoking	3,486 (21.68)	945 (27.11)	2,541 (72.89)		
Quit smoking	1,107 (6.88)	318 (28.73)	789 (71.27)		
Non-smoking	11,487 (71.44)	3,410 (29.69)	8,077 (70.31)		
Chronic conditions				313.471	<0.001
No	12,465 (77.52)	4,048 (32.47)	8,417 (67.53)		
Yes	3,615 (22.48)	625 (17.29)	2,990 (82.71)		
Self-rated health status				439.361	<0.001
Good	4,814 (29.94)	1,602 (33.28)	3,212 (66.72)		
Relatively good	5,377 (33.44)	1890 (35.15)	3,487 (64.85)		
Average	4,899 (30.47)	1,091 (22.27)	3,808 (77.73)		
Relatively poor	691 (4.30)	64 (9.26)	627 (90.74)		
Poor	299 (1.86)	26 (8.70)	273 (91.30)		

### HL level of residents in Anhui Province

**Figure 2:**
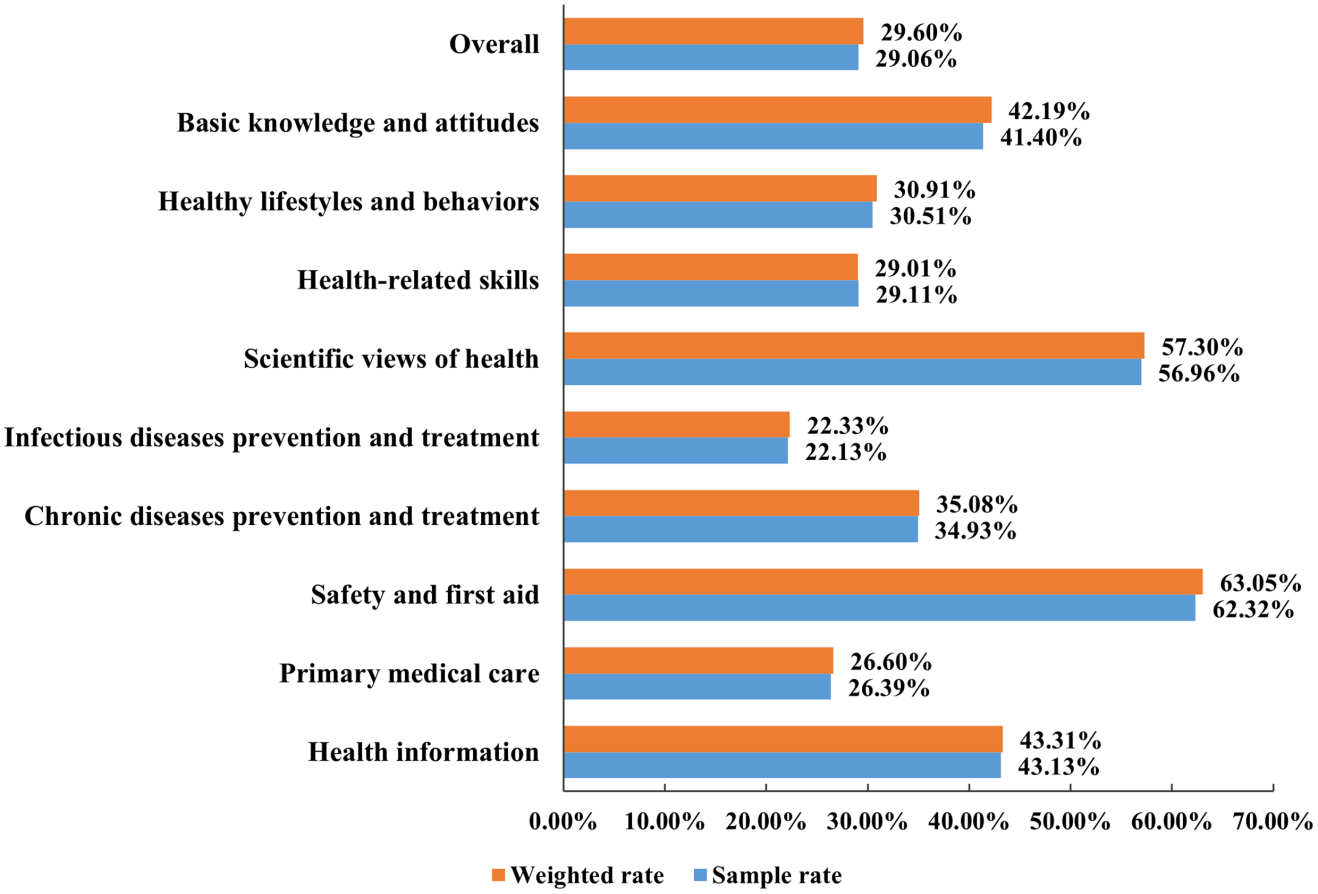
The proportion of respondents with adequate HL by three dimensions and six aspects.

In this survey, 29.06% (4,673/16,080) of the respondents had adequate HL. After weighted adjustment, the level of HL of Anhui Province was 29.60%. The level of HL in three dimensions: BKA level was 42.19%, HLB level was 30.91%, and HRS level was 29.01%. Additionally, for the six aspects of HL, from high to low, were as follows: safety and first aid (63.05%), scientific views of health (57.30%), health information (43.31%), chronic diseases prevention and treatment (35.08%), primary medical care (26.60%), and infectious diseases prevention and treatment (22.33%) (Figure 2).

**Figure 3:**
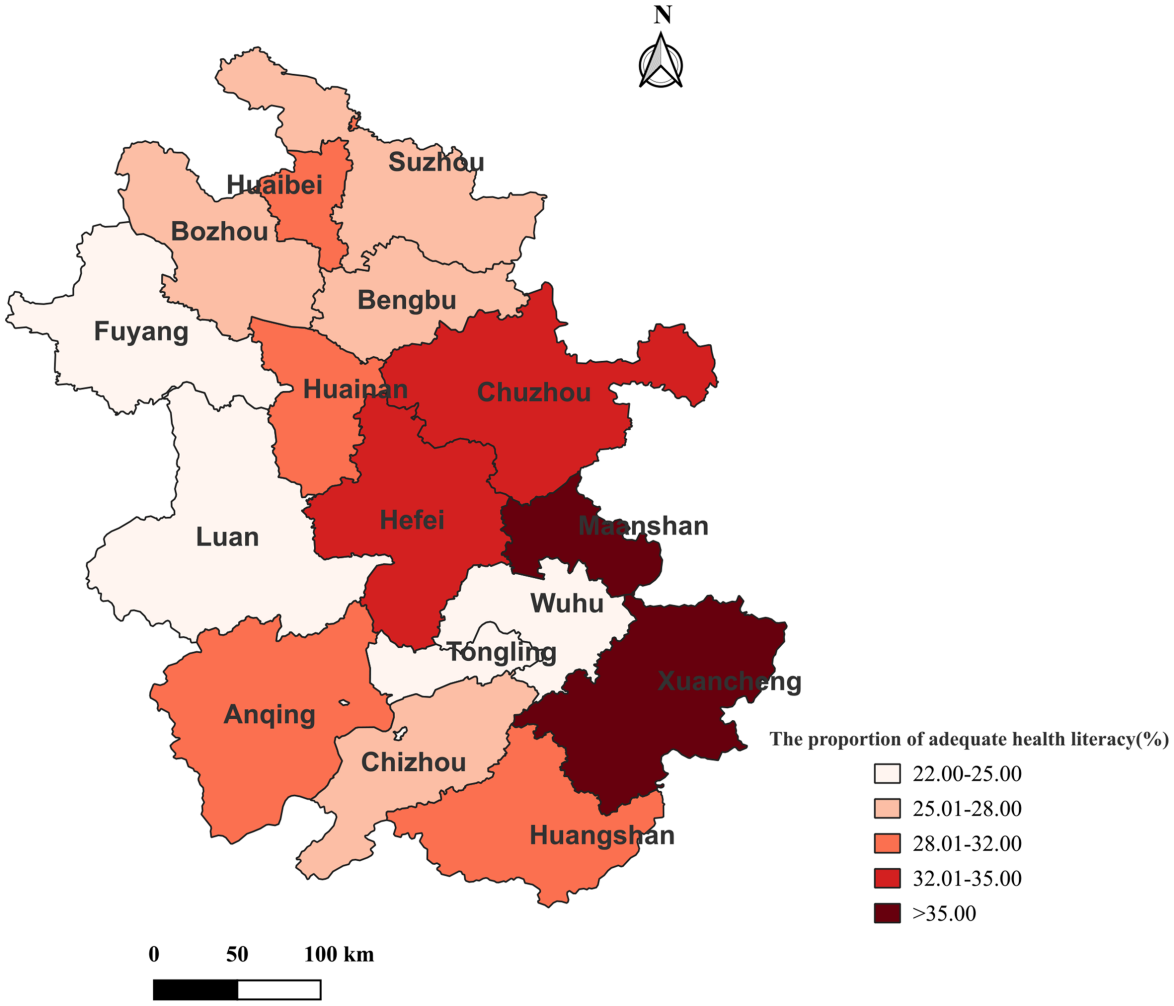
The proportion of respondents with adequate HL in different cities of Anhui Province.

Figure 3 shows the geographic distribution of cities by the proportion of participants with adequate HL. The results indicate that the HL level in the central and southern regions of Anhui Province was slightly higher than that in the northern regions, except for Tongling and Wuhu. Furthermore, the proportion of adequate HL ranged from 38.73% (Xuancheng) to 22.42% (Tongling).

### Adequate HL and demographic characteristics

Chi-squared test was performed to compare the level of HL among different demographic groups (Table 1). Statistically significant differences were observed among the following groups: gender (*χ*2 = 44.514, *p* < 0.001), age group (*χ*2 = 1,206.792, *p* < 0.001), education level (*χ*2 = 282.212, *p* < 0.001), occupation (*χ*2 = 1,411.747, *p* < 0.001), marital status (*χ*2 = 307.914, *p* < 0.001), smoking status (*χ*2 = 8.682, *p* = 0.013), chronic condition (*χ*2 = 313.471, *p* < 0.001), and self-rated health status (*χ*2 = 439.361, *p* < 0.001). No statistically significant difference was found in place of residence (*χ*2 = 0.111, *p* = 0.739) and ethnicity (*χ*2 = 2.407, *p* = 0.121).

### Factors associated with adequate HL

In univariate analysis, gender, age group, education level, occupation, marital status, smoking status, chronic condition, and self-rated health status were significantly associated with adequate HL.

**Figure 4:**
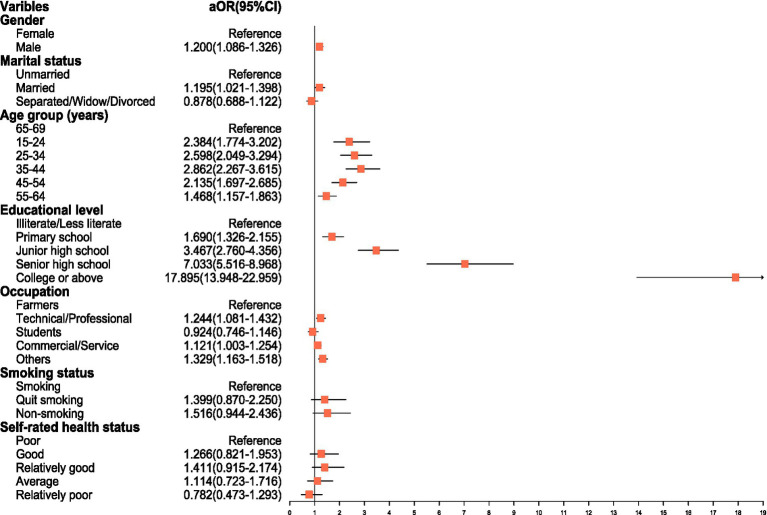
Multivariable logistic regression analysis of factors associated with adequate health HL.

All variables associated with adequate HL in univariate analyses were included in a multivariable logistic regression model. The results revealed that, compared with females, males were more likely to have adequate HL (aOR = 1.200; 95% CI: 1.086–1.326). Married individuals were more likely to have adequate HL compared to unmarried individuals (aOR = 1.195, 95% CI: 1.021–1.398). Compared with illiterate/less literate, the aOR values for primary school, junior high school, senior high school, and college or above were 1.690 (1.326–2.155), 3.467 (2.760–4.356), 7.033 (5.516–8.968), and 17.895 (13.948–22.959), respectively, indicating that higher education levels were correlated with higher HL levels. Compared with the age group of 65–69 years, the aOR values for the age groups 15–24, 25–34, 35–44, 45–54 and 55–64 years were 2.384 (1.774–3.202), 2.598 (2.049–3.294), 2.862 (2.267–3.615), 2.135 (1.697–2.685), and 1.468 (1.157–1.863), respectively. In addition, compared with farmers, the aOR values were 1.244 (95% CI, 1.081–1.432) for technical/professional, 1.121 (95% CI, 1.003–1.254) for commercial/service, and 1.329 (95% CI, 1.163–1.518) for other occupations (Figure 4).

## Discussion

HL is a comprehensive evaluation indicator reflecting the health level of the people, and improving HL can reduce the unfairness of health services and social costs (21). In this study, the primary objective were to assess the HL status among urban and rural residents in Anhui Province and to identify the key factors associated with HL. The novelty is further underscored by the detailed examination of HL across different demographic groups within a single province, offering a granular view not previously available. The results revealed that the overall HL rate in Anhui Province was 29.60%, which is slightly higher than the national average (27.78%) (22), but still relatively low compared to some developed countries, such as the United States and Japan (23, 24). Furthermore, compared with other developed provinces in China, such as Beijing (40.5%) (25) and Shanghai (38.25%) (26) the HL level of Anhui residents was still at a low level. This indicates that the HL level of residents in a region is related to economic and social development. In addition, it also suggests that the HL level of residents in Anhui Province must to be improved urgently.

Among the three dimensions of HL, the level of BKA was the highest, which was higher than the levels of HLB and HRS, which is in line with the results of the studies conducted by Zhang et al. (27) and Rong et al. (18). This demonstrates that after acquiring health knowledge and concepts, the residents did not effectively transform them into health behaviors and skills, and there was a certain degree of “knowledge-action inconsistency.” Based on the Knowledge-Attitude-Practice (KAP) theoretical model (28), behavioral change in individuals requires three processes: acquiring knowledge, generating beliefs, and forming behaviors. Therefore, it is suggested that in future health education efforts, targeted health education and promotion activities should be implemented based on the primary factors associated the formation of residents’ healthy behaviors, aiming to maximize the promotion of healthy behaviors and cultivate health-related skills.

The analysis of six aspects of HL revealed that the level of safety and first aid was the highest, which may be related to the frequent occurrence of public health emergencies in recent years, such as the COVID-19 pandemic. On the one hand, the government has continuously increased its efforts in emergency response and widely disseminated safety and first aid knowledge and skills. On the other hand, the public’s awareness of safety and first aid has been increasing, and more attention has been paid to the acquisition and utilization of safety and first aid knowledge. It is worth noting that among the six aspects of HL, the level of infectious diseases prevention and treatment was the lowest, at only 22.33%, which is similar to the results of a survey conducted in 25 provinces in China (29). The possible reason is that in the past work on improving HL, more attention may have been paid to the popularization of health knowledge and skills in other aspects, while the emphasis on infectious diseases prevention and treatment was relatively insufficient. Since infectious diseases not only cause harm to individuals, but also may seriously affect public health and cause huge economic burdens (30). Improving the level of infectious diseases prevention and treatment can not only effectively prevent and control the occurrence and spread of infectious diseases, but also avoid blind panic and discrimination caused by lack of relevant knowledge (31). Therefore, in the coming period of time, enhancing the level of infectious diseases prevention and treatment among residents in Anhui Province will be a key area for carrying out health education and promotion programs.

This study found a significant association between adequate HL and age. With the increase of age, the level of HL shows a trend of gradually decreasing, which is consistent with previous studies (4, 32, 33). The possible reason was that young people have strong learning abilities, and adept at utilizing various new media and social channels to acquire health-related knowledge, medical, and health service information, and were also more likely to adopt healthy lifestyles and skills. In contrast, the memory of the older adults gradually declines, and their interest in learning new knowledge and skills also decreases. Furthermore, their access to health information and knowledge is relatively limited, and many of their beliefs and behavioral habits were already ingrained, making it difficult to change. According to data from the National Bureau of Statistics of China, by the end of 2023, the older adult population aged 60 and above in China was approximately 297 million, accounting for 21.1% of the total population (34). With the acceleration of China’s aging process, the health issues of the older adults were receiving increasing attention, and improving the HL of the older adults is an important strategy and measure to improve their health status and promote active aging (35, 36). Therefore, in the future, the older adult population will be a key focus in improving HL.

The results of this study revealed that HL was strongly associated with education level. A higher education level was independently associated with a higher level of HL, which is consistent with previous research findings (37–39). People with lower education levels exhibit lower abilities in learning and comprehending health knowledge and information, weaker health awareness, and less experience in interacting with health professionals than the general population (40). Conversely, those with higher education levels possess stronger reading comprehension and self-management skills, as well as higher health awareness and skills, enabling them to better utilize medical service resources (41). Consequently, when designing health education interventions, it is imperative to enrich the content and format of health education tailored to different educational groups. Specifically, for those with lower education levels, a balance must be struck between authority and acceptability, with a primary focus on methods that are more illustrated and less written, vivid and interesting, and adapted to their comprehension abilities.

Multivariable logistic regression analysis also showed that gender, occupation, and marital status were associated with the level of HL. In this study, the proportion of males with adequate HL was higher than that of females, which differed from the results of previous studies (29, 42, 43). A possible explanation of this finding is that the overall education level of males in this survey is higher than that of females. In addition, in many rural areas of Anhui Province, there is a phenomenon of favoring boys over girls, which leads to different roles and expectations of males and females in society, which in turn affects their attitudes and behaviors toward health, making males more inclined to take active health management measures. Among all occupations, farmers’ HL was significantly lower than that of technical/professional and others. Possible reasons include the generally lower educational attainment of farmers, which limits their ability to learn and understand health knowledge. Second, farmers have limited access to and utilization of medical resources, as well as limited channels for participating in health education activities (44). Interestingly, our findings reveal that unmarried individuals had a lower level of HL than married individuals. This may be attributed to the fact that married individuals often assume greater familial responsibilities, such as caring for children and the older adults. Such a sense of responsibility may motivate them to pay more attention to their own health status, thereby enabling them to better fulfill their familial roles.

There are several limitations to this study. First, due to the differences in the definition and measurement tools of HL in different countries, it is difficult to directly compare the HL levels of different countries. Second, the participants in this study were only collected from 38 monitoring sites in Anhui Province, which may limit the generalizability of the findings. Future studies should consider conducting research in a broader geographical area to validate the findings of this study. Finally, a further limitation is the cross-sectional design, which means that cause-effect conclusions could not be drawn.

## Conclusion

In summary, the HL level of residents in Anhui Province was relatively low, we recommend that policymakers in Anhui Province prioritize HL improvement programs targeting specific subpopulations, such as females, the older adults, farmers, and individuals with low educational attainment. These programs should focus on developing tailored health communication models and educational materials that are culturally appropriate, easily understandable, and engaging. Additionally, establishing community-based initiatives, leveraging digital platforms for health information dissemination, and fostering partnerships with healthcare providers and community organizations can further enhance HL levels. Continuous monitoring and evaluation of these programs will be crucial to adjust strategies and ensure their effectiveness.

## Data Availability

The raw data supporting the conclusions of this article will be made available by the authors, without undue reservation.
